# The Frequency Effect of the Motor Imagery Brain Computer Interface Training on Cortical Response in Healthy Subjects: A Randomized Clinical Trial of Functional Near-Infrared Spectroscopy Study

**DOI:** 10.3389/fnins.2022.810553

**Published:** 2022-03-31

**Authors:** Qiang Lin, Yanni Zhang, Yajie Zhang, Wanqi Zhuang, Biyi Zhao, Xiaomin Ke, Tingting Peng, Tingting You, Yongchun Jiang, Anniwaer Yilifate, Wei Huang, Lingying Hou, Yaoyao You, Yaping Huai, Yaxian Qiu, Yuxin Zheng, Haining Ou

**Affiliations:** ^1^Department of Rehabilitation, The Fifth Affiliated Hospital of Guangzhou Medical University, Guangzhou, China; ^2^Fifth Clinical School, Guangzhou Medical University, Guangzhou, China; ^3^Department of Rehabilitation, Guangzhou Key Laboratory of Enhanced Recovery After Abdominal Surgery, The Fifth Affiliated Hospital of Guangzhou Medical University, Guangzhou, China; ^4^Department of Rehabilitation Medicine, Shenzhen Longhua District Central Hospital, Shenzhen, China

**Keywords:** functional near infrared spectroscopy, cortical response, frequency effect, motor imagery, brain computer interface

## Abstract

**Background:**

The motor imagery brain computer interface (MI-BCI) is now available in a commercial product for clinical rehabilitation. However, MI-BCI is still a relatively new technology for commercial rehabilitation application and there is limited prior work on the frequency effect. The MI-BCI has become a commercial product for clinical neurological rehabilitation, such as rehabilitation for upper limb motor dysfunction after stroke. However, the formulation of clinical rehabilitation programs for MI-BCI is lack of scientific and standardized guidance, especially limited prior work on the frequency effect. Therefore, this study aims at clarifying how frequency effects on MI-BCI training for the plasticity of the central nervous system.

**Methods:**

Sixteen young healthy subjects (aged 22.94 ± 3.86 years) were enrolled in this randomized clinical trial study. Subjects were randomly assigned to a high frequency group (HF group) and low frequency group (LF group). The HF group performed MI-BCI training once per day while the LF group performed once every other day. All subjects performed 10 sessions of MI-BCI training. functional near-infrared spectroscopy (fNIRS) measurement, Wolf Motor Function Test (WMFT) and brain computer interface (BCI) performance were assessed at baseline, mid-assessment (after completion of five BCI training sessions), and post-assessment (after completion of 10 BCI training sessions).

**Results:**

The results from the two-way ANOVA of beta values indicated that GROUP, TIME, and GROUP × TIME interaction of the right primary sensorimotor cortex had significant main effects [GROUP: *F*_(1,14)_ = 7.251, *P* = 0.010; TIME: *F*_(2,13)_ = 3.317, *P* = 0.046; GROUP × TIME: *F*_(2,13)_ = 5.676, *P* = 0.007]. The degree of activation was affected by training frequency, evaluation time point and interaction. The activation of left primary sensory motor cortex was also affected by group (frequency) (*P* = 0.003). Moreover, the TIME variable was only significantly different in the HF group, in which the beta value of the mid-assessment was higher than that of both the baseline assessment (*P* = 0.027) and post-assessment (*P* = 0.001), respectively. Nevertheless, there was no significant difference in the results of WMFT between HF group and LF group.

**Conclusion:**

The major results showed that more cortical activation and better BCI performance were found in the HF group relative to the LF group. Moreover, the within-group results also showed more cortical activation after five sessions of BCI training and better BCI performance after 10 sessions in the HF group, but no similar effects were found in the LF group. This pilot study provided an essential reference for the formulation of clinical programs for MI-BCI training in improvement for upper limb dysfunction.

## Introduction

In recent years, brain computer interface (BCI) technology has matured into a potentially helpful tool. BCI technology establishes a direct real-time connection between the brain and external devices without relying on peripheral nerves or muscles to achieve human-computer interaction (Mane et al., 2020). There are different types of BCI, one of which is based on motor imagery (MI), called motor imagery-BCI (MI-BCI). This form of BCI is now available in a commercial product for the clinical rehabilitation of upper limb motor dysfunction after stroke, and has achieved positive results (Chaudhary et al., 2016).

Motor imagery brain computer interface converts the generated motor intention of the subject’s motor imagery into motor instructions. It thus commands external devices such as robots to perform actual movement. It can also generate corresponding tactile, visual, and proprioceptive feedback, thus forming a central-peripheral-central active closed-loop control system (Potter et al., 2014). The MI-BCI system achieves repeated recruitment of motor neurons circuit during training to promote neural plasticity, thus repairing connections between damaged neurons and ultimately improving motor dysfunction. Floriana Pichiorri and Mattia (2020) found that compared with simple MI training, the Fugl-Meyer assessment scores of hemiplegic upper limbs in hospitalized patients with subacute stroke recovery and severe motor dysfunction using MI-BCI were significantly increased. Similar results can be seen in chronic stroke patients with severe hand weakness (Ramos-Murguialday et al., 2013).

Previous studies have also shown that active and repetitive reinforcement of functional activity is important for nerve remodeling and motor function recovery (Mane et al., 2020). Therefore, clarifying how the duration, frequency, and intensity of MI-BCI training affects the plasticity of the central nervous system and clinical function is crucial for developing MI-BCI rehabilitation programs. However, there is limited prior work on the frequency effect. Other neuromodulation studies have shown a correlation between neural plasticity and training frequency. Bai et al. (2020) found that repetitive transcranial magnetic stimulation (rTMS) training twice a day was more effective than once a day in promoting neuroplasticity in the language area of the brain, repairing or enhancing the connection between related neurons, and improving the language function of patients with aphasia after stroke. Accordingly, we assumed that there may also be a frequency effect in MI-BCI.

Current studies of the neural plasticity changes related to BCI mostly use the following neuroimaging methods: functional magnetic resonance imaging (fMRI) (Zhang et al., 2021), electroencephalography (EEG) (Abiri et al., 2019), and functional near-infrared spectroscopy (fNIRS) (Yang et al., 2019). Of these, fNIRS is reflects the neural activity of the brain indirectly via real-time monitoring of the concentration changes in hemoglobin and deoxyhemoglobin in the cerebral cortex under different stimulation tasks. Versus fMRI, fNIRS can be used in real environments for real-time monitoring. It is simple to use and has high temporal resolution. fNIRS has higher spatial resolution and is less affected by the head movement of subjects (Yang et al., 2019). Therefore, fNIRS has high application potential in the field of neuromodulation rehabilitation. Ding et al. (2021) found that BCI training improved brain functional connectivity between motor cortex and prefrontal cortex via fNIRS. Kaiser et al. (2014) also determined that MI-BCI training increased the cortical activation of the supplementary motor cortex (SMA) and the primary motor cortex. It also enhanced event-related desynchronization (ERD) through fNIRS testing.

However, MI-BCI is still a relatively new technology for commercial rehabilitation applications. It is crucial to understand the MI-BCI frequency effect for clinical standardized treatment. The heterogeneity of stroke patients is high and includes age and cognitive function: These both affect the therapeutic effect of MI-BCI. At the same time, because of the less use and less flexibility of the non-handedness (compared with the handedness), it was used to simulate the hemiplegic upper limbs of stroke patients and handedness stimulates the unaffected side. In this study, we evaluated the effect of MI-BCI frequency on the cortical function of non-handedness, and these numerical controls could help guide clinical application and future MI-BCI research. Therefore, to minimize the impact of the subject heterogeneity on the results, our study recruited young healthy subjects with right-handedness to reduce the effects of brain injury in different regions and degrees as well as the effects of age, cognitive function, motor function, and lateralization of the brain. We also used non-handedness to simulate the improvement of upper limb function on the hemiplegic side to evaluate the regulation effect of MI-BCI frequency on cortical function of non-handedness. This data can help guide clinical applications and future MI-BCI research.

## Materials and Methods

### Participants

This study recruited 22 young healthy subjects (age, 22.36 ± 3.53; males were 31.82%) from Guangzhou Medical University who met the following criteria: (a) right handedness as assessed by the Edinburgh Handedness Inventory; (b) no history of neurological diseases; (c) no history of brain and upper limb trauma, and (d) no cognitive impairment. Participants were excluded if they had one of the following exclusion criteria: (a) medications that reduce seizure thresholds or psychotropic medications; (b) any personal factors affected the EEG signal of the BCI leading to instability or making it impossible to collect fNIRS data; or (c) unable to finish the whole experimental. This study was approved by the Ethics Committee of the Fifth Affiliated Hospital of Guangzhou Medical University (No. KY01-2021-05-01) and had international clinical trial registration (ChiCTR2100050162).^1^ All participants signed informed consent forms.

### Study Design

**FIGURE 1:**
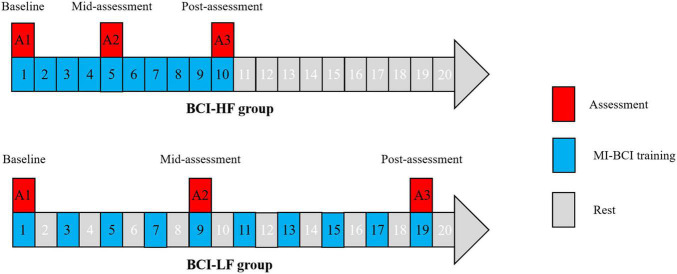
Motor imagery brain computer interface (MI-BCI) training and assessment design in the High Frequency and Low Frequency groups.

In this randomized clinical trial (RCT) study, 22 subjects were randomly assigned to a high frequency group (HF group) and low frequency group (LF group) in a 1:1 ratio using computer-generated random numbers. The HF group performed BCI training once per day while the LF group performed once every other day. All subjects proceeded over 10 sessions in BCI training (30 min for one session) and assessed the clinical assessment and fNIRS testing at three time points: baseline, mid-assessment (after completion of five BCI training sessions), and post-assessment (after completion of 10 BCI training sessions) (Figure 1).

### Intervention

**FIGURE 2:**
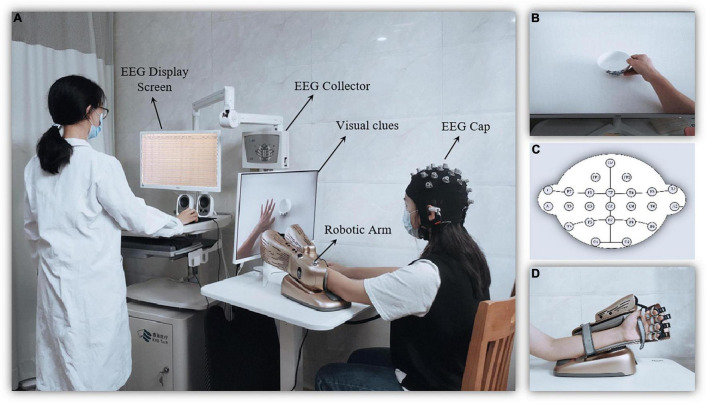
Diagram of the motor imagery brain computer interface (MI-BCI) upper limb rehabilitation training system. **(A)** The MI-BCI training setting. **(B)** The screen providing visual clues for motor imagery. **(C)** The robotic arm for motion performing and feedback. **(D)** Electroencephalography (EEG) electrode placement.

Non-dominant hand function training was performed by the MI-BCI system (BCI-Hand with 24 EEG channels, Rehab Medical Technology Co., Ltd., Shenzhen, China), which consists of an EEG cap, a computer terminal (i.e., the control interface), an external manipulator, and a 23-inch computer monitor (Figure 2A). Subjects performed motor imagery by watching video cues about hand function (Figure 2B). The EEG cap was based on the International 10–20 System as a reference (Figure 2C), with 24-electrode conduction channels (including 22 recording electrodes and 2 reference electrodes) setting over the frontal and parietal regions. The EEG data were collected using the EEG amplifier with unipolar Ag/Ag-cl electrode channels, digitally sampled at 256 Hz with a 22-bit resolution for voltage ranges of ±130 mV. The real-time EEG signals collected were amplified by computer terminal according to the central processing control algorithm, and the mu ERD score (score from 0 to 100) during motor imagery was calculated. The external robotic arm would be driven when the score reaches 60 points. The non-dominant hand could perform the action while providing real-time feedback (both sensory and visual) (Figure 2D).

### Preparation

Subjects were instructed on the experimental process and arranged in a comfortable sitting position. They were asked to minimize physical activity during BCI training. The researchers put EEG caps onto the subjects, instructed them to remain relaxed, and adjusted Electrodes to maintain waveform of the EEG signal smooth. The sampling frequency of the EEG system is 10–100 Hz. Finally, investigators placed manipulators on the subjects’ non-dominant hand and adjusted them to be comfortable and ensure that they did not slip out during training.

### Motor Imagery Brain Computer Interface Training

Motor imagery brain computer interface training included baseline acquisition phase and training phase. During the baseline acquisition phase, subjects were instructed to remain relaxed and collect a stable baseline EEG signal for 1 min. During the training phase, subjects performed 30 min of motor imagery tasks followed by pre-set video prompts on the computer screen. Each run was composed of one motor imagery task and one relaxation task. There were then 10 runs in one trial, and 6 to 8 trials were required for one session. The number of trials was mainly affected by the completion of the motor imagery task, and the difficulty of completing the task required more time.

Motor imagery tasks had various levels of difficulty. Different levels of difficulty had various requirements for motor imagery. The initial difficulty was set as 13 referring to the level of healthy subject. Upon completion of the last task, the system adjusted the difficulty level of the next task. The initial difficulty was set to 13. Each run has three chances to complete the MI task at this level per trial. If subjects failed to complete the MI task for three times, then the trial was considered a failure, and the difficulty level was automatically decreased in the next trial. If subjects completed all 10 runs in one trial, then the difficulty level was automatically upgraded in the next trial. The motor imagery tasks involved in the trial were all hand grasping motions, which could be divided into two categories: grasping and opening hand. Grasping action including but not limited to book, toothbrush, cups, chess, rubber, keys, etc. With the upgrading of difficulty, grasping objects tend to be small and exquisite.

### Clinical Functional Assessment

The Wolf Motor Function Test (WMFT) was used to assess bilateral upper limb motor function with 17 items in total (Morris et al., 2001). Items 1–8 were used to assess isolated movement of the shoulder and elbow, and items 9–17 were used to assess the overall upper limb movement (shoulder, elbow and hand). Items 7 and 14 were strength measurements and only recorded the corresponding value but not the movement quality. The remaining items were scored in terms of movement quality using a 6-point scale (0 = does no attempt; 5 = normal movement) for a total of 75 scores. The ratio of grip strength was calculated based on Item 14 as the strength of non-dominant/left hand divided by the strength of dominant/right hand.

### Brain Computer Interface Performance

The BCI performance was calculated via the MI task difficulty level and score using the specific formula: [Trial 1 (difficulty level × average score) + Trial 2 (difficulty level × average score) + … + Trial *n* (difficulty level × average score)]/the number of difficulty levels. Here, “*n*” is the number of trials completed for each session.

### Functional Near-Infrared Spectroscopy Measurement and Data Processing

**FIGURE 3:**
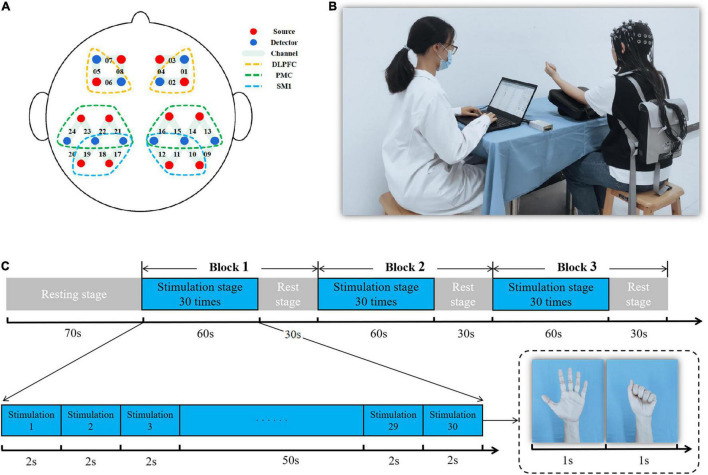
Diagram of the functional near-infrared spectroscopy (fNIRS) measurement. **(A)** Regions of interest and the channel setting; **(B)** fNIRS measurement presentation; **(C)** fNIRS experimental paradigm.

A 24-channel fNIRS device (Nirsmart, Danyang Huichuang Medical Equipment Co., Ltd., Jiangsu, China) was used with setting source probes and detectors according to a 10–20 system. Two source probes and two detectors were placed on the left and right frontal lobes, respectively. Four source probes and three detectors were placed on the left and right parietal lobes, respectively. The channel setting is shown in Figure 3A. We recorded data with wavelengths of 730 and 850 nm. The fNIRS device converts the optical signals into the concentration changes of oxygenated hemoglobin (HbO_2_) and deoxygenated hemoglobin (HbR) according to the modified Beer-Lambert Law to investigate the effects of different stimulus conditions on cortical activation (Pinti et al., 2020).

The motor task paradigm of fNIRS was a non-dominant grasping task at a frequency of 0.5 Hz that included a 70-s rest stage and a 270-s stimulation stage for 340 s of fNIRS testing. Of these, the stimulation stage consisted of three trials (60-s stimulation and 30-s rest for one trial) (Figures 3B,C).

There were six regions of interest: bilateral primary sensorimotor cortex (SM1), bilateral promotor cortex (PMC), and bilateral dorsolateral prefrontal cortex (DLPFC). Bilateral SM1 were covered by channels 10, 11, 12, 17, 18, and 19. These mainly included primary somatosensory cortex (S1) and primary motor cortex (M1) and achieved motor learning through sensory and motor input (Gomez et al., 2021). Bilateral PMC were covered by channels 13, 14, 15, 16, 21, 22, 23, and 24 and involved motor planning (Li et al., 2015). Bilateral DLPFC were covered by channels 1, 2, 5, and 6 and were mainly responsible for cognitive, emotional, and sensory processing (Seminowicz and Moayedi, 2017; Figure 3A). The average value of the channels that overlapped more than 50% in the regions-of-interest were used as an outcome value of the cortex (Wan et al., 2018).

The original data collected by fNIRS were pre-processed by NIRSPARK software including artifact processing, filtering, segmentation, and baseline comparison. We converted optical density into blood oxygen concentration data—these data were block averaged and statistically analyzed to calculate the beta values for the region-of-interest. Thus, the differences of the cortical activation from GROUP (HF group and LF group) and TIME (baseline, mid-assessment, and post-assessment) could be compared. The general linear model (GLM) for was used to estimate of the hemodynamic response at individual-level fNIRS data statistical analysis individual-level statistical analysis. For GLM specification, the canonical hemodynamic response function was used to construct the reference time series representation from task variables. The estimation of GLM parameters on a channel-by-channel basis, which calculated the activation beta value (weight coefficient in the linear model) for each experimental condition (Hou et al., 2021).

### Statistical Analysis

Statistical analysis used SPSS25.0 software. Measurement data confirmed a normal distribution via mean ± standard deviation; count data were represented by rate or constituent ration. Two independent sample *t*-tests were used to compare the baseline measurement data between the two groups including homogeneity of variance and normal distribution. The parameters (beta values, BCI performance, and WMFT scores) of GROUP effect (HF and LF), TIME effect (baseline, mid-assessment and post-assessment) and GROUP × TIME interaction effects were analyzed by two-way analysis of variance. *Post-hoc* tests used multiple comparison Bonferroni corrections. Statistical significance was defined as *P* < 0.05.

## Results

**FIGURE 4:**
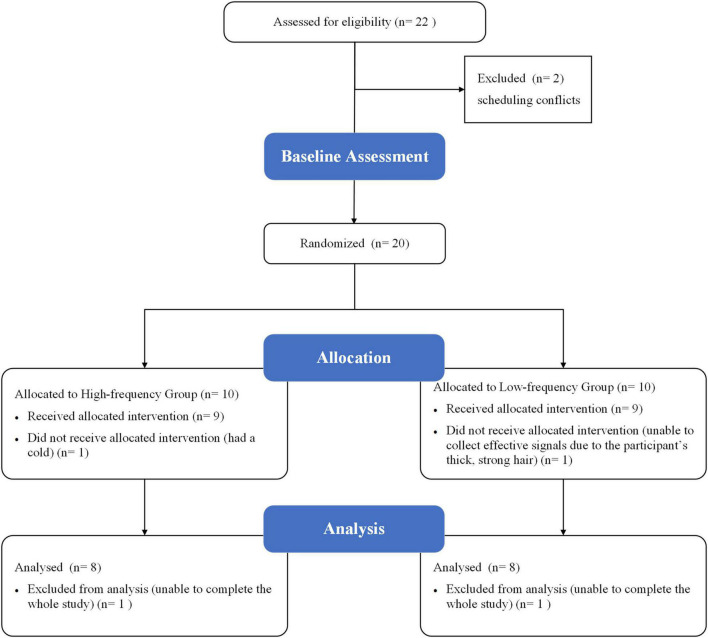
Enrollment diagram.

This study originally enrolled 22 participants; however, only 16 completed all MI-BCI training and assessments. Of the six participants who dropped out of the study, two were attributed to scheduling conflicts for the baseline assessment; one was unable to collect effective signals from fNIRS due to the participant’s thick, strong hair; one was due to having a cold during MI-BCI training; and the remaining two were unable to complete the whole study. No participant reported any adverse events or results during the MI-BCI training and assessments (Figure 4). Ultimately, 16 subjects (aged 22.94 ± 3.86 years; 31.25% males) were equally randomly assigned to either the HF or LF group. At baseline, no significant differences were found in age, sex ratio, WMFT scores, BCI performance, and beta values of ROIs between the two groups (all *P* > 0.05).

The results from the two-way ANOVA of WMFT scores and the grip strength ratio showed no significant main effect in GROUP, TIME, and GROUP × TIME interaction. The results from the two-way ANOVA of MI-BCI performance revealed a significant main effect [*F*_(1,16)_ = 8.210, *P* = 0.006] in frequencies. However, no significant main effect was found for TIME, and GROUP × TIME interaction (Table 1).

**TABLE 1:** Results of analysis of variance (ANOVA) conducted on GROUP, TIME, and interaction effect on wolf motor function test (WMFT) and brain computer interface (BCI) performance.

Assessment indicators	Main effect (GROUP)		Main effect (TIME)		Interaction effect (GROUP*TIME)	
			
	F	*P*-values	F	*P*-values	F	*P*-values
**WMFT scores**						
Dominant/right hand	2.000	0.165	0.500	0.610	0.500	0.610
Non-dominant/left hand	2.032	0.161	1.581	0.218	0.677	0.513
**The ratio of grip strength (%)**	2.704	0.108	0.689	0.508	0.390	0.680
**BCI performance (scores)**	8.210	**0.006**	0.549	0.582	0.069	0.934

*GROUP factor refers to the combination of the high frequency and low frequency groups. TIME factor refers to baseline, mid-assessment, or post-assessment. The ratio of grip strength was calculated as the strength of the non-dominant/left hand divided by the strength of the dominant/right hand. The calculation formula of BCI performance is described in the methodology section. P-values less than 0.05 indicate statistically significant differences and are marked in bold.*

*WMFT, Wolf motor function test; BCI, brain computer interface.*

**FIGURE 5:**
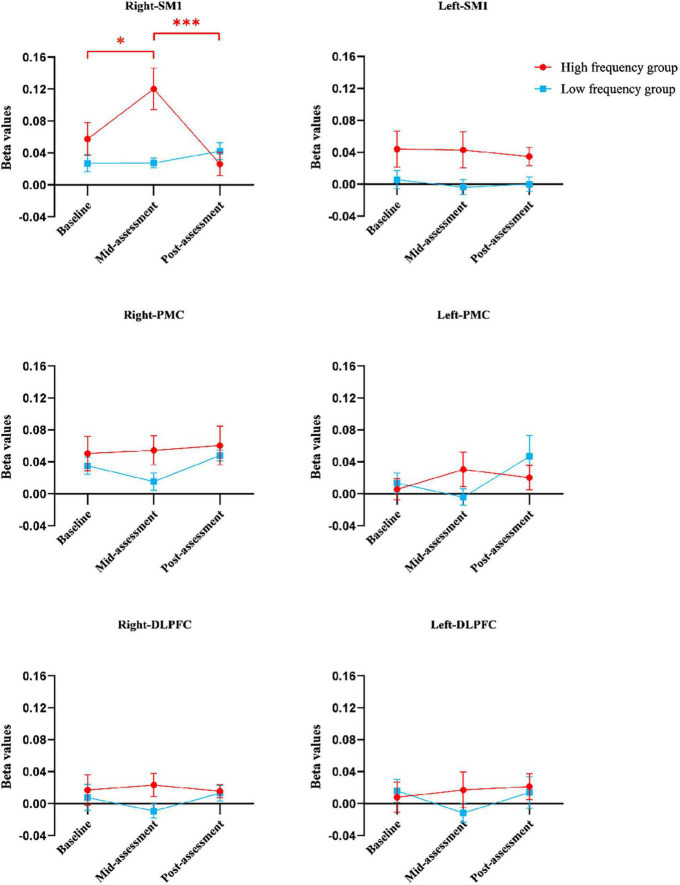
The beta values of regions of interests in different groups at three assessment time-points. Error bars represent standard errors. *Indicates statistical significance *P* < 0.05 and ***indicates statistical significance *P* < 0.001. DLPFC, dorsolateral prefrontal cortex; PMC, promoter cortex; SM1, primary sensory-motor cortex.

**FIGURE 6:**
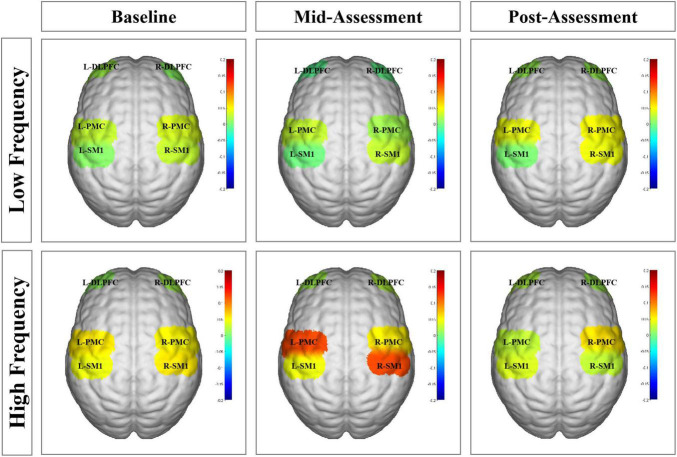
Functional near-infrared spectroscopy (fNIRS) activation maps in different groups at three assessment time-points. The beta values are indicated by color. L-DLPFC, left dorsolateral prefrontal cortex; R-DLPFC, right dorsolateral prefrontal cortex; L-PMC, left promoter cortex; R-PMC, right promoter cortex; L-SM1, left primary sensory-motor cortex; R-SM1, right primary sensory-motor cortex; L, left; R, right.

The results from the two-way ANOVA of beta values in ROIs indicated that GROUP, TIME, and GROUP × TIME interaction of the right SM1 had significant main effects [GROUP: *F*_(1,14)_ = 7.251, *P* = 0.010; TIME: *F*_(2,13)_ = 3.317, *P* = 0.046; GROUP × TIME: *F*_(2,13)_ = 5.676, *P* = 0.007] (Table 2 and Figures 5, 6). The *post-test* results showed a significant difference between the groups at mid-assessment (*P* < 0.001) (Table 3). Moreover, the TIME variable was only significantly different in the HF group, in which the beta value of the mid-assessment was higher than that of both the baseline assessment (*P* = 0.027) and post-assessment (*P* = 0.001), respectively (Table 4). The beta value trend of the baseline assessment was higher than that of the post-assessment; however, this result was not statistically significant. The two-way ANOVA of the left SM1 results showed that only GROUP variable had a significant main effect [*F*_(1,14)_ = 9.849, *P* = 0.003] and that no significant main effect was found for TIME and GROUP × TIME interaction (Table 1). The *post-test* revealed that significant differences were only found between groups in the mid-assessment (*P* = 0.040) (Table 3). The two-way ANOVA of bilateral PMC and DLPFC results revealed no significant main effect of GROUP, TIME, and GROUP × TIME interaction (Table 1).

**TABLE 2:** Results of analysis of variance (ANOVA) conducted on GROUP, TIME, and interaction effect for beta values of regions of interest.

Regions of interest	Main effect (GROUP)		Main effect (TIME)		Interaction effect (GROUP*TIME)	
			
	*F*	*P*-values	*F*	*P*-values	*F*	*P*-values
**SM1**						
Right	7.251	**0.010**	3.317	**0.046**	5.676	**0.007**
Left	9.849	**0.003**	0.123	0.884	0.077	0.926
**PMC**						
Right	2.704	0.108	0.689	0.508	0.390	0.680
Left	0.000	0.994	1.096	0.344	1.597	0.215
**DLPFC**						
Right	1.810	0.186	0.154	0.857	0.680	0.512
Left	0.422	0.519	0.363	0.698	0.552	0.580

*GROUP factor refers to the combination of the high frequency and low frequency groups. TIME factor refers to baseline, mid-assessment, or post-assessment. P-values less than 0.05 indicate statistically significant differences and are marked in bold.*

*SM1, primary sensorimotor cortex; PMC, primary motor cortex; DLPFC, dorsolateral prefrontal cortex.*

**TABLE 3:** The beta values of SM1 in the Bonferroni correction for multiple comparisons between high frequency group and low frequency group.

TIME	Right SM1			Left SM1		
		
	Mean difference	Standard error	*P*-values	Mean difference	Standard error	*P*-values
Baseline	0.030	0.023	0.195	0.038	0.022	0.089
Mid-assessment	0.093	0.023	**<0.001**	0.047	0.022	**0.040**
Post-assessment	−0.016	0.023	0.488	0.035	0.022	0.122

*TIME factor refers to baseline, mid-assessment, or post-assessment. P-values less than 0.05 indicate statistically significant differences and are marked in bold.*

*SM1, primary sensorimotor cortex.*

**TABLE 4:** The beta values of SM1 in the Bonferroni correction for multiple comparisons within high frequency group and low frequency group, respectively.

GROUP	Right SM1			Left SM1		
		
	Mean difference	Standard error	*P*-values	Mean difference	Standard error	*P*-values
**High frequency group**						
Baseline vs. Mid-assessment	-0.063	0.023	**0.027**	0.001	0.022	1.000
Baseline vs. Post-assessment	0.031	0.023	0.547	0.009	0.022	1.000
Mid-assessment vs. Post-assessment	0.094	0.023	**0.001**	0.008	0.022	1.000
**Low frequency group**						
Baseline vs. Mid-assessment	0.000	0.023	1.000	0.010	0.023	1.000
Baseline vs. Post-assessment	-0.015	0.023	1.000	0.006	0.023	1.000
Mid-assessment vs. Post-assessment	-0.015	0.023	1.000	-0.004	0.023	1.000

*GROUP factor refers to the combination of the high frequency and low frequency groups. P-values less than 0.05 indicate statistically significant differences and are marked in bold.*

*SM1, primary sensorimotor cortex.*

## Discussion

In recent years, the MI-BCI system based on the closed-loop control theory has become a research hotspot due to its great potential application prospect in rehabilitation filed for upper limb dysfunction caused by the central nervous system (Ang and Guan, 2015). However, no relevant guideline is available on the clinical parameter setting of this novel technology (Mane et al., 2020), which restricts BCI clinical application. Our study used fNIRS to explore the frequency effect of MI-BCI training for non-dominant hand functions and cortical activation in normal subjects. To our knowledge, this study is the first RCT on the frequency-response of the MI-BCI upper limb rehabilitation system. In this study, all subjects in the HF and LF groups received ten sessions of non-dominant hand MI-BCI training. The clinical evaluation results of WMFT were not affected by the training frequency. The possible reason was that in order to exclude the heterogeneity of the subjects, the selected subjects were all healthy young individuals, and the upper limbs were not affected, and there was not much room for improvement. Therefore, the clinical evaluation of Wolf failed to reflect the subtle changes in the upper limb function of the subjects. Although no statistically significant differences were found between these two groups in WMFT performance, the fNIRS evaluation results showed that frequency (GROUP effect) presented a main effect on the contralateral SM1 activation. Furthermore, compared with baseline values, contralateral SM1 activation increased in the HF group after five consecutive sessions of BCI training. Meanwhile, BCI performance in the HF group was better than that in LF group after 10 consecutive sessions of BCI training. Several possible explanations and mechanisms are presented below.

First, brain neuroplasticity, including the reorganization of brain structure and function, occurs throughout the human lifespan (Dimyan and Cohen, 2011). Meanwhile, a previous study has confirmed the positive correlation between functional improvement after stroke and enhanced neuroplasticity following rehabilitative interventions (Draaisma et al., 2020). The BCI system based on the closed-loop principle is used to compensate for the absent feedback information due to peripheral limb motor dysfunction by external devices (Potter et al., 2014), such as robotic arms, visual feedback system, or functional electrical stimulation (FES). Therefore, BCI training has aroused new interest for rehabilitative purposes—especially for patients with moderate-to-severe motor dysfunction who are limited to other conventional treatment measures (Young et al., 2014). Moreover, previous small sample BCI studies have also shown positive effect for stroke patients not only in the subacute stage, but also in the chronic stage (Cervera et al., 2018). However, given that the clinical application of BCI remains relatively new, the effect of frequency as one of the important parameters has not yet been studied. Among relevant publications to date, the frequency of BCI training was inconsistent. For example, some studies scheduled BCI training at a frequency of twice per week (Johnson et al., 2018), while others had a seven time-per-week schedule (Nishimoto et al., 2018). Considering the critical recovery and neural plasticity stage spanning the first 6 months post-stroke (Hendricks et al., 2002), great significance must be placed on clarifying the frequency parameter setting of BCI training to maximize rehabilitation and the ultimate functional outcomes. In order to eliminate the heterogeneity in patient subjects and explore in isolation the frequency effect of BCI training on neuroplasticity, young and healthy subjects were enrolled to perform non-dominant hand MI-BCI training in our study. The cortical response was found to be more visible after 5 sessions of BCI training in the HF group, but not in the LF group. On the other hand, other clinical BCI training studies on stroke patients found functional improvement after BCI training at a frequency of five times per week (Ramos-Murguialday et al., 2013; Mukaino et al., 2014). Based on the perspectives of cortical modulation and functional improvement, all of the results indicated the potential of HF BCI training to yield positive effects, which may constitute important references for future treatment in patient populations. Nevertheless, Young et al. mainly explored the dose-response on BCI training and incidentally involved in the frequency effect. The relevant results of this clinical retrospective study with a small sample size suggested no significant difference in frequency effect, which was inconsistent with the results of our study (Young et al., 2015). Two major reasons were considered, one of which may be related to the differing populations studied (stroke patients recruited in the study by Young et al.; normal subjects recruited in our study). The other reason may be related to the differing total number and frequency of BCI training sessions for each subject in Young’s study. Furthermore, in the study by Young et al., LF treatment was defined as ≤2 times per week, whereas HF treatment was defined as >2 times per week, in which the range of HF group was considerably broad (Young et al., 2015). For example, the three times per week schedule identified as HF in the study by Young et al. was still considered as the LF BCI intervention in our study. Therefore, additional studies on frequency response in patients are needed. Furthermore, the MI-BCI training procedure requires relatively high cognitive ability (Carelli et al., 2017). For example, sufficient cognitive ability is needed to understand the content of BCI training and to cooperate with the demands of the MI task. Furthermore, attention must be sustained over the 30-min training session. All participants recruited for our study were college students enrolled in Guangzhou Medical University. The heterogeneity of such samples, including cognitive and attention levels, has been well-controlled. However, in real clinical settings, the patient’s cognition and attentional capacity are not only affected by various diseases, but also by other confounding factors such as age and education level. Thus, future research is needed to better delineate the frequency effects of BCI training in different populations. In addition, after ten sessions of MI-BCI training, the BCI performance of the HF group in our study was improved relative to the LF group. The BCI performance score was considered more related to the MI performance and attentional level during the training sessions. Previous studies have reported that BCI training could improve motor function and cognitive function concurrently (Ali et al., 2020). Furthermore, BCI has also been designed for use in children with attention deficit hyperactivity disorder (ADHD) (Qian et al., 2018). We also found improvement in BCI performance in the HF group relative to the LF group, which suggested that HF training may be more beneficial to cognitive improvement than LF training. These findings could also be used to guide the formulation of future clinical BCI training programs.

Finally, compared within HF group, more contralateral cortical activation was found after five training sessions than in baseline data, whereas no difference was found after ten training sessions. Moreover, as shown in Figure 5A, the beta value of right/contralateral SM1 was the highest after five-session training and then decreased back to baseline levels after ten training sessions in HF group. A possible explanation might be that non-dominant gripping is considered as relatively simple to master for healthy, young subjects. So, the increased contralateral SM1 activation from baseline to after five training sessions might involve in a process of neural recruitment during motor learning, whereas the decreased contralateral SM1 activation seen between the 5- to the 10-session training might involve in motor acquisition, which indicated that the brain operated in the most economical tendency (Paparella et al., 2020). However, although this decrease was seen in the HF group, it was not seen in the LF group, which also suggested that HF MI-BCI training may have a greater potential on motor relearning than LF MI-BCI training. Thus, MI-BCI training tasks whose difficulty level can be tailored should be considered for clinical application, and corresponding MI-BCI modules should be generated for improved clinical rehabilitation.

### Limitation

This study was a pilot study of frequency-response for the MI-BCI training system. There were two main limitations to this study. First, only healthy young subjects were included in an effort to control for heterogeneity. Future studies should extend research populations to include, for example, healthy elderly subjects or stroke patients with varying degrees of brain damage and functional levels to further explore frequency-response and provide more evidence for guiding clinical application. Second, this study only explored a single type of external MI-BCI equipment (i.e., a robotic arm) for providing feedback in the MI-BCI system. Future research efforts are encouraged to assess different external equipment, such as virtual reality and FES, and explore different frequency effects based on comprehensive factors.

## Conclusion

In this study, healthy young participants underwent ten sessions at varying frequencies of MI-BCI training on non-dominant hand function. The results showed that more cortical activation and better BCI performance were found in the HF group relative to the LF group. Moreover, the within-group results also showed more cortical activation after five sessions of BCI training and better BCI performance after ten sessions in the HF group, but no similar effects were found in the LF group. These results indicated that a 30-min session duration once per day for five consecutive days may be the minimum effective dose of MI-BCI training for evoking cortical activation modulation in healthy subjects, which could be deduced to the population with central nervous system disease, such as stroke patients, in the future. This pilot RCT study provides an important theoretical basis for the clinical application of MI-BCI training for improving upper limb dysfunction.

## Data Availability Statement

The raw data supporting the conclusions of this article will be made available by the authors, without undue reservation.

## Ethics Statement

The studies involving human participants were reviewed and approved by the Ethics Committee of the Fifth Affiliated Hospital of Guangzhou Medical University. The patients/participants provided their written informed consent to participate in this study.

## Author Contributions

HO, YxZ, YQ, and QL designed the study. QL, YnZ, and YJZ drafted the manuscript. WZ, BZ, and XK performed data analysis. TP, TY, and YJ wrote sections of the manuscript. AY drew the figures. WH, LH, YY, and YH collected the data. HO, YxZ, and YQ approved the final version of the manuscript. All authors contributed to manuscript revision, read, and approved the submitted version.

## Conflict of Interest

The authors declare that the research was conducted in the absence of any commercial or financial relationships that could be construed as a potential conflict of interest.

## Publisher’s Note

All claims expressed in this article are solely those of the authors and do not necessarily represent those of their affiliated organizations, or those of the publisher, the editors and the reviewers. Any product that may be evaluated in this article, or claim that may be made by its manufacturer, is not guaranteed or endorsed by the publisher.

## Footnotes

1
https://www.chictr.org.cn
